# A Novel Biomimetic Nanoprobe as a Photoacoustic Contrast Agent

**DOI:** 10.3389/fchem.2021.721799

**Published:** 2021-08-03

**Authors:** Xin Huang, Ao Shen, Rui Peng, Sheng Chen, Shitao Lin, Shangwei Ding, Huan Li, Dazhi Zhou

**Affiliations:** ^1^ The First Affiliated Hospital of Guangzhou Medical University, Guangzhou, China; ^2^ Key Laboratory of Molecular Target and Clinical Pharmacology and the State Key Laboratory of Respiratory Disease, School of Pharmaceutical Sciences, Guangzhou Medical University, Guangzhou, China; ^3^ Institute of Orthopedic Diseases and Center for Joint Surgery and Sports Medicine, The First Affiliated Hospital, Jinan University, Guangzhou, China; ^4^ Department of Neurosurgery, Xinqiao Hospital, Army Medical University, Chongqing, China

**Keywords:** photoacoustic contrast agent, nanoprobe, targeted therapy, poly(D,L-lactic-co-glycolic acid), photoacoustic imaging

## Abstract

Specific detection of tumors is of pivotal importance to cancer prevention and therapy yet a big challenge. Photoacoustic imaging (PAI) as an emerging non-invasive modality has shown great potential in biomedical and clinical applications. The performance of PAI largely depends on the light-absorption coefficient of the imaged tissue and the PAI contrast agent being used, either endogenously or exogenously. The exogenous contrast agents developed so far have greatly helped to improve PAI, but still have some limitations, such as lack of targeting capacity and easy clearance by the host immune system. Herein, we fabricated a biomimetic nanoprobe with cell membrane coating as a novel PAI contrast agent, namely, MPD [membrane-coated poly(lactic-co-glycolic acid) (PLGA)/dye]. In brief, the organic dye 1,1′-dioctadecyl-3,3,3′,3′-tetramethylindotricarbocyanine iodide (DiR) was encapsulated by the Food and Drug Administration–approved polymer, poly(lactic-co-glycolic acid) (PLGA), to form polymer nanoparticles by emulsification. The nanoparticles are further coated with the cancer cell membrane to form MPD. MPD has outstanding biocompatibility, tumor specificity, and *in vivo* stability. Thus, MPD is a versatile NIR-I theranostic nanoplatform for PAI-guided cancer diagnosis and therapy.

## Introduction

Imaging tests are of vital importance in clinical decision-making. Currently, multiple imaging procedures are being widely used in cancer diagnosis, therapy, and follow-up. In many cases, a small amount of radiation is required, such as X-rays, positron emission tomography (PET), computed tomography (CT), magnetic resonance imaging (MRI), and single-photon emission computed tomography (SPECT) (Lee et al., 2012). Although well-developed and reliable, these imaging strategies still have their limitations (Franc et al., 2008; Liu et al., 2012). Photoacoustic imaging (PAI) is a fast-developing non-radiation modality with a unique imaging principle that transverses the pulsed laser signal absorbed by tissue to the ultrasonic signal after calculation by certain arithmetic. PAI offers rich optical contrasts, high ultrasound spatial resolution, and deep penetration depth (Li et al., 2020). PAI thus becomes a promising, efficient, and precise imaging strategy for the evaluation of cancers.

PAI takes advantage of the features mentioned above by direct optical absorption. PAI benefits from contrast agents that generate a characteristic signal which is different from the background. In biological tissues, both endogenous and exogenous contrast agents can be exploited as imaging targets. Some commonly used contrast agents include endogenous hemoglobin and melanin, exogenous optical dyes, small organic molecules, liposomes, and nanoparticles (NPs) (Wang et al., 2006; Wang et al., 2018; Hariri et al., 2019; Upputuri and Pramanik, 2020). However, some limitations remain in current contrast agents. For example, dyes lack sensitivity and targeting capacity; NP-based contrast agents are easy to be cleared by the host immune system, resulting in low imaging efficiency. Thus, developing novel probes to overcome current obstacles is demanding.

Recently, biomimetic functionalization engineering NPs have shown superior biocompatibility and smart targeting to desired tissues (Zhang et al., 2020a; Peng et al., 2021). Specifically, biomimetic NPs with cell membrane coating manifest cell-material hybrid nanoplatforms that combine the advantages of both natural and synthetic elements (Kroll et al., 2017; Zhang et al., 2020b). Biomimetic NPs own special functions because they have the protein of the source cells, such as ligand recognition and targeting (Chen et al., 2020), immune escaping (Hu et al., 2011), and long blood circulation (Gao et al., 2017).

Many types of cell membranes (such as red blood cells, cancer cells, and immune cells) coating NPs with incredible functions and features have been prepared (Li et al., 2018). To name a few examples, these include leukocyte membrane–cloaked silica microparticles with endothelium traversing properties (Chang et al., 2020), platelet membrane–coated nanovesicles with cancer-targeting capabilities (Dehaini et al., 2017), and red blood cell membrane–cloaked NPs capable of performing long circulation (Chen et al., 2019).

A good PAI contrast agent needs many important features, including but not limited to high molar-extinction coefficient, near-infrared (NIR) peak absorption, excellent photostability, low toxicity and immunogenicity, high target affinity and specificity, and high biocompatibility (Chong et al., 2018). We developed a novel biomimetic nanoprobe with cell membrane coating as a photoacoustic contrast agent, namely, MPD (membrane-coated PLGA/dye) nanoparticles. MPD can target the tumor tissue precisely and prolong the calculation time in the blood due to the immune escape ability of the cancer cell membrane. In brief, DiR dye is encapsulated by PLGA to form polymer nanoparticles by emulsification, and the nanoparticles are further coated with the cancer cell membrane to form MPD. MPD has outstanding biocompatibility, tumor specificity, and *in vivo* stability. Thus, MPD is a versatile NIR-I theranostic nanoplatform for PAI-guided cancer diagnosis and therapy.

## Results and Discussion

### Synthesis and Characterization of MPD

**FIGURE 1:**
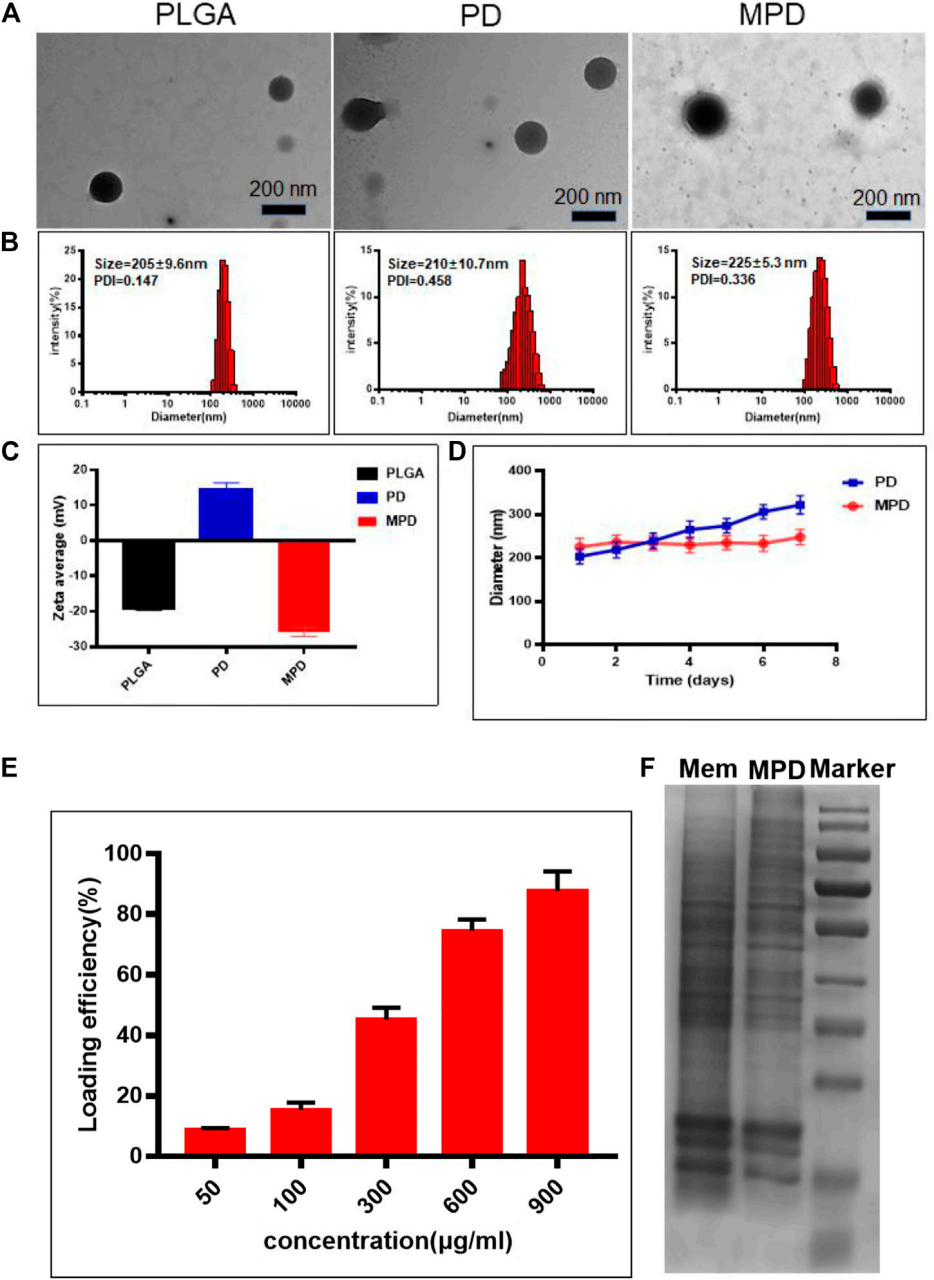
Characterization and degradation of PLGA-based nanoparticles. **(A)** TEM image of different nanoparticles. Scale bar: 200 nm. **(B)** DLS analysis of PLGA-based nanoparticles. **(C)** Surface zeta potential of PLGA-based nanoparticles (*n* = 3). **(D)** Stability analysis of PLGA and MPD (*n* = 3). **(E)** Loading capacity assessment of MPD with different concentrations of DiR (*n* = 3). **(F)** SDS-PAGE analysis of the surface proteins on MPD. PD, PLGA/DiR, MPD, LLC membranes/PLGA/DiR.

PLGA NPs, PLGA NPs with DiR dye (PD), and MPD NPs were prepared according to the details described in Methods. Transmission electron microscopy analysis demonstrated that all three types of NPs had a solid and spherical form (Figure 1A). Dynamic light scattering (DLS) showed that the sizes of PD were approximately 210 ± 10.7 nm (Figure 1B). The MPD was camouflaged with a thin film layer, and the sizes of MPD were 225 ± 5.3 nm, which was 10–15 nm thicker than PD (Figures 1A,B). Furthermore, the zeta potential of PLGA NPs was about −20 mV, while that of PD changed to a positive charge after loading with DiR. Finally, the zeta potential of MPD became −23.5 mV after modifying with the cell membrane isolated from mouse Lewis lung cancer (LLC) cells. The change of zeta potentials indicated that PD was coated with negative LLC membrane successfully (Figure 1C). We performed UV-visible absorption spectroscopy to analyze the MPD and found that both absorbances of PLGA and DiR are shown in the spectrum (Supplementary Figure 1). Particularly, MPD had a uniform size distribution in water, and no distinct size variations were found after 7 days, while PD had an obvious size increase after 4 days (Figure 1D). These results demonstrated that MPD had good stability after modifying with the LLC membrane. The phenomenon is attributed to some reasons: 1) the absolute value of MPD was ∼25 mV, about 10 mV higher than that of PD; 2) the camouflage with cell membranes lowered the surface energy; and 3) the camouflage with cell membranes reduced the aggregation or non-specific adsorption. The characteristics were also confirmed by the previous work (Zhang et al., 2020b). Furthermore, we measured the DiR loading capacities with increasing DiR concentrations (Figure 1E). The loading capacity was as high as 82% when DiR concentration was 900 μg/ml, which indicated that the drug loading capacity could meet the requirement of the *in vivo* experiment. To confirm the proteins of the LLC cell membrane retain on the surface of MPD, sodium dodecyl sulfate–polyacrylamide gel electrophoresis (SDS-PAGE) was conducted. SDS-PAGE showed that lane 1 is similar to lane 2, which indicated that MPD kept the whole proteins of the LLC cell membrane (Figure 1F).

### *In Vitro* Cytotoxic Effect of MPD

**FIGURE 2:**
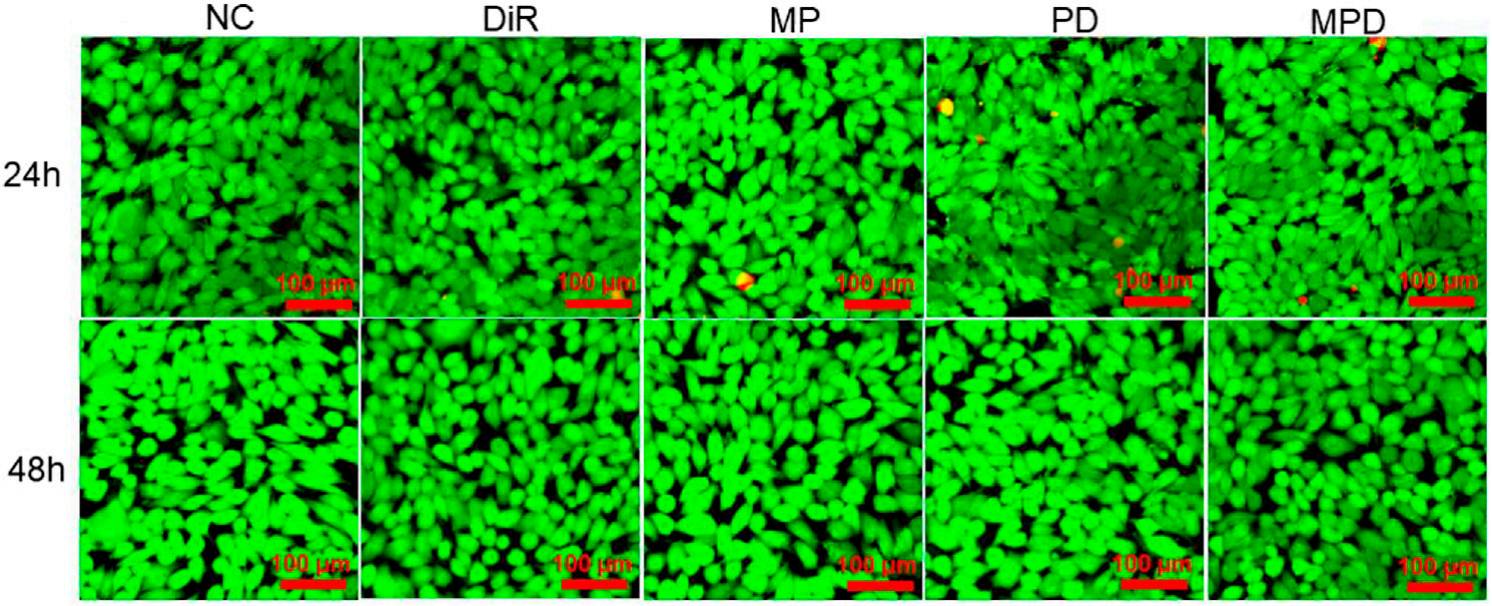
Cytotoxic assay of LLC cells treated with MPD and different control formulations.

Subsequently, we assessed the biocompatibility of MPD by using calcein-AM and PI to stain the living and dead cells, respectively. The results showed that the LLC cell viability was similar after 24 and 48 h incubation with MPD (Figure 2). Furthermore, the control groups, such as NC, DiR, LLC membrane/PLGA (MP), and PD, showed no apparent toxicity in 24 and 28 h. The cell viability was also evaluated by Cell Counting Kit-8 (CCK-8), which was consistent with that evaluated by the Live/Dead assay (Supplementary Figure 2).

## Cell Uptake by PAI

**FIGURE 3:**
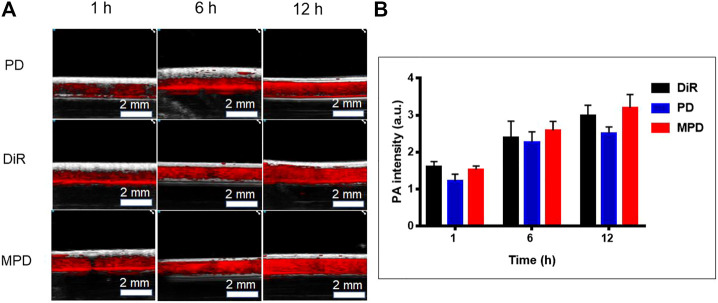
*In vitro* photoacoustic imaging analysis. **(A)** PA image of DiR-based nanoparticles at different time points. **(B)** Quantitative analysis of DiR-based nanoparticles at different time points (*n* = 3). LLC cells were seeded to the cell culture dishes with a density of 5 × 10^5^ cells/dish for 24 h. After incubation for 1, 6, and 12 h with free DiR or nanocomposite materials (PD and MPD) containing the same amount of DiR, respectively, the cells were collected and fixed with paraformaldehyde. The cell suspension was put into the imaging capillary tube and sealed at both ends with a lighter. The LAZR-X multimode imaging system was used to detect the PA signals. The Vevo LAB 3.2.0 software was applied to analyze data and fluorescence intensity.

The cell uptake of DiR-based nanoparticles was observed by a Vevo LAZR-X photoacoustic imaging system. The result showed that the PA signal showed a time-dependent increase of DiR. Several photoacoustic signals were observed after 1 h incubation with the DiR-based nanoparticles. With the increase of time, the cellular uptake increased, which was indicated by the increased photoacoustic signal after 12 h incubation. As shown in Figure 3, the fluorescence of MPD was the same as that of the pure DiR group and 1/4-fold higher than that of the PD group at the same time. Noticeably, the membrane-modified nanoparticles promoted their uptake by cells. This phenomenon may be caused by receptor-mediated mechanisms on the cell membrane.

## *In Vivo* PAI Capability

**FIGURE 4:**
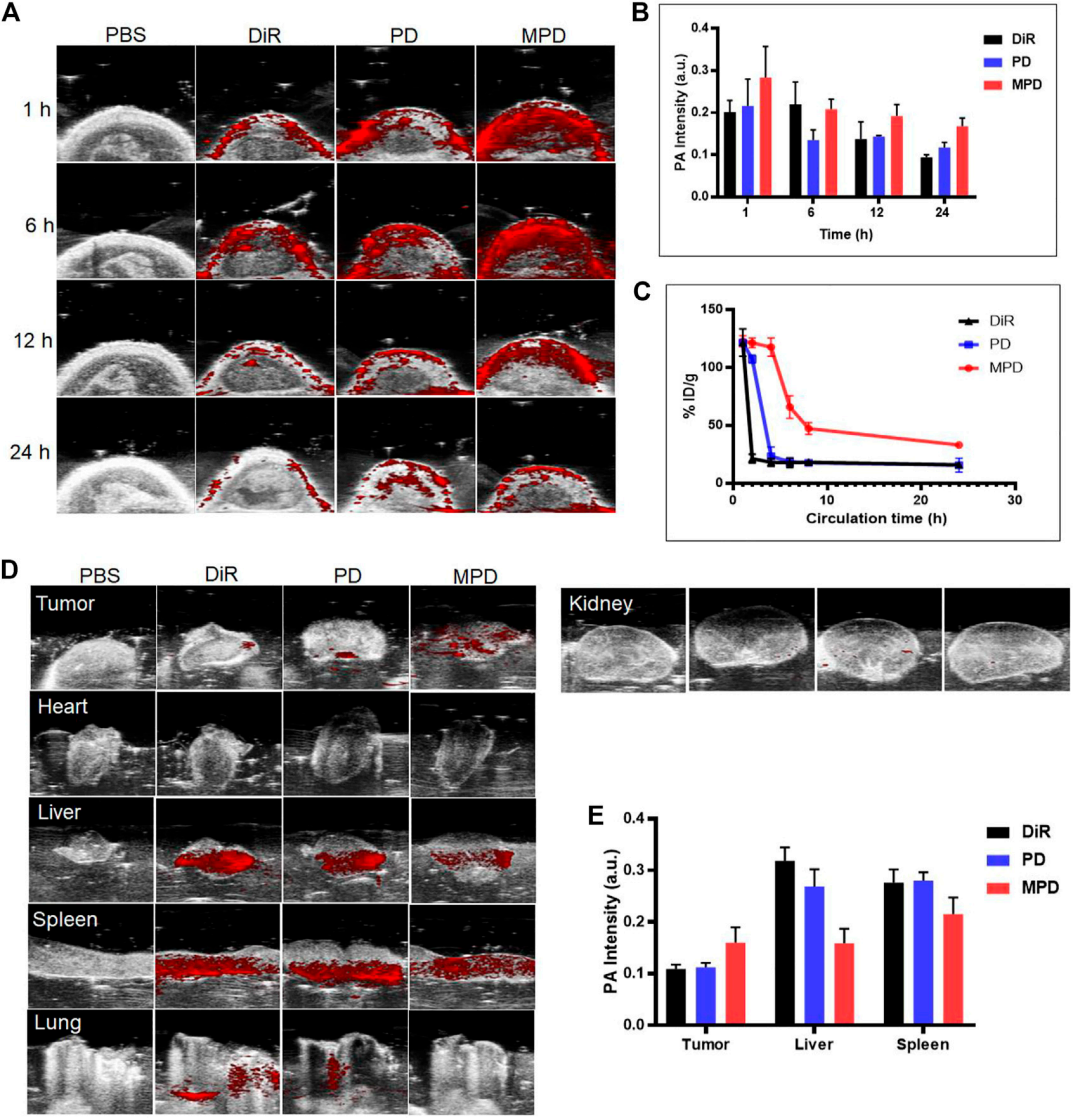
*In vivo* photoacoustic imaging. **(A)** MPD was administered to the mice. PA imaging was applied to analyze the distribution of MPD at different time points (1, 6, 12, and 24 h). **(B)** Quantitative analysis of MPD distribution in tumor sites at different time points (1, 6, 12, and 24 h) (*n* = 3). **(C)** Fluorescence signal detection of blood retention of MPD (*n* = 3). **(D)** PA image of major organs and tumors after 24 h. **(E)** Quantitative analysis of MPD distribution of major organs and tumors after 24 h (*n* = 3). The tumor-bearing mice were administered with the same amount of saline (200 μL), DiR (200 μL, 1 mg/ml), and PD and MPD containing the same amount of DiR through the tail vein. At 1, 4, 8, and 24 h after injection, the PA signals were monitored by the Vevo LAZR-X multimode imaging system using the Step and Shoot modes with 100 angles and 15 pulses per angle. Mice were then sacrificed 24 h after injection to check the bioelimination in different organs and tumors. Data were analyzed and quantified by Vevo LAB 3.2.0 software. For the evaluation of the circulation lifetime, the tumor-bearing mice were injected with saline, DiR, and PD and MPD containing the same amount of DiR through the tail vein. At 1, 2, 4, 6, 8, and 24 h after injection, the DiR content in the blood was collected and analyzed with a multifunctional microplate reader (Tecan Infinite Pro, Switzerland).

The PAI ability of MPD was investigated and compared with that of DiR and PD in living mice bearing xenograft LLC tumors. After intravenous injection of MPD, PD, or DiR, we monitored the PA signals at 1, 6, 12, and 24 h. The PA images at different time points were recorded and quantified under the pulse laser excitation at 715 nm. After the organs of mice were harvest after the 24 h PA imaging time point, we analyzed the PA signal of each organ. As shown in Figure 4A, the PA intensities gradually accumulated at tumor sites and reached their maxima at 6 h after injection. At this time point, the PA intensity of MPD was 1/2-fold higher than that of PD- and DiR-treated groups (Figure 4B). We also detected the circulation lifetime *in vivo*, and found that the MPD showed a much longer circulation lifetime, compared with the control groups, such as DiR and PD (Figure 4C). Thus the coating with cancer cell membranes improved the circulation lifetime significantly. Furthermore, when the PA signals of individual organs were quantified, PA intensity was most pronounced in the liver, spleen, and tumor sites (Figure 4D). Particularly, the strongest PA signals were captured in tumors of MPD-treated mice. The PA signal accumulation in the liver and spleen was less than that observed in PD- and DiR-treated groups, which indicated that the LLC membrane–modified nanoparticles kept the homotypic targeting ability of the cancer cell membrane (Figure 4E).

## Conclusion

In summary, we have developed a novel biomimetic nanoprobe MPD loaded with DiR and with cell membrane coating as a photoacoustic contrast agent, which assisted NIR-I diagnosis for cancer. MPD shows great physiological stability, excellent biocompatibility, and superior PAI properties that allow for deep tissue imaging *in vivo*. MPD displays a great ability of tumor targeting and long circulation time in the blood. The design of membrane-modified nanoparticles has great advantages in different types of cancer PAI diagnostics. As a result, we firmly believe that MPD may have significant potential in clinical settings in the future.

## Methods

In this study, PLGA/DiR nanoparticles were prepared by the double emulsification solvent evaporation method. The biomimetic nanoprobe (MPD) loaded with DiR was obtained by encapsulating the cell membrane by the ultrasonic method. The particle size and morphology were characterized by transmission electron microscopy, and particle size, zeta potential, and stability were investigated by a laser particle size analyzer. The entrapment efficiency and drug loading were investigated by fluorescence detection. Capillary photoacoustic imaging was used to investigate the effect of cell imaging *in vitro*, and tumor photoacoustic imaging was used to investigate the accumulation and imaging effects *in vivo* and the retention-time changes in the blood of nanocomposite mice.

## Preparation of PLGA, PD, and MPD Nanoparticles

### Preparation of PLGA Nanoparticles

The nanoparticles were prepared by the double emulsification solvent evaporation method: 10 mg PLGA was weighed into a 10 ml centrifuge tube. A mixed solution of 700 μL dichloromethane and 300 μL ethyl acetate was added and then dissolved in the water bath for 10 min as the oil phase (O). 1 ml of ultra-pure water was taken as an aqueous phase (W) in another centrifugal tube, and the aqueous phase was added to the oil phase drop by drop. At the same time, the colostrum was formed by intermittent ultrasound in the ice bath under the ultrasonic pulverizer for 5 min with a power of 80 W and 2/1 s on/off cycles. The colostrum was then added drop by drop to 3% polyvinyl alcohol (PVA) aqueous solution (5 ml) and sonicated with a power of 80 W and 2/1 s on/off cycles for 5 min. The obtained double emulsion was added to 0.3% PVA aqueous solution (10 ml) and stirred magnetically for 3 h at room temperature and pressure until the organic solvent evaporated completely. Then, PLGA nanoparticles were obtained by high-speed centrifugation (4°C, 12,000 rpm, and 40 min), washed with ultra-pure water for three times, and finally resuspended with 1.0 ml ultra-pure water or freeze-dried and stored at −20°C.

### Preparation of PD Nanoparticles

DiR was dissolved in dimethyl sulfoxide (DMSO) and added into the oil phase prepared as described in Preparation of PLGA Nanoparticles at the mass ratio of 7:1 (PLGA:DiR). After being uniformly mixed in the water bath and ultrasonicated for 10 min, the following steps were the same as those in Preparation of PLGA Nanoparticles to obtain PD nanoparticles. This step was carried out in the dark.

### Preparation of MPD Nanoparticles

The membrane (M) of mouse Lewis lung cancer (LLC) cells was isolated and extracted according to the instructions of the cell membrane protein and cytoplasmic protein extraction kit (Biyuntian, China). PD and M were mixed at the mass ratio of 1:2, vortexed for 3 min, ultrasonicated for 30 min, and continuously extruded 11 times with a micro-extruder (Avanti, United States) to obtain MPD. This step was carried out in the dark.

## Investigation of Related Characteristics of MPD Nanosystem

### Determination of Particle Size, Zeta Potential, Dispersion Coefficient, and Stability

Each sample was diluted with ultra-pure water at a rate of 1:9 to ensure that the surface charge is measured accurately at low ionic strength. After dilution, the final concentration of the sample to be tested is 0.1 mg/ml. The particle size (size), dispersion coefficient (PDI), and zeta potential (surface charge) of PLGA, PD, and MPD were measured by ZetaSizer (Malvern, Worcestershire, United Kingdom) at room temperature. The particle size, PDI, and zeta potential were measured at a series of specific time points (1, 2, 3, 4, 5, 6, and 7 days).

### Transmission Electron Microscope Observation

PLGA, PD, and MPD solutions will be prepared according to the above procedures. A total of 10 μL of the samples was taken with a pipette and dropped onto the copper net coated with a carbon film. After standing for 3 min, the excess liquid was removed with filter paper and left at room temperature for 1 min. Then, a drop of ultra-pure water was dropped on the sealing film, and then the copper mesh face was buckled down on the ultra-pure water droplets. After standing for 2 min, the filter paper was used to absorb the excess ultra-pure water. After drying, observation was carried out under a transmission microscope, and pictures were taken.

### Analysis of Loading Capacity

#### Determination of PLGA Load Rate and Encapsulation Rate

As mentioned above, four kinds of PLGA/DiR nanoparticles with different mass ratios (1:1/3:1/5:1/7:1) were obtained by fixing the feeding amount of DiR and changing the feeding amount of PLGA. The nanocomposite PLGA/DiR, loaded with DiR, was collected by centrifugation, and the concentration of DiR in the supernatant was determined by a full-wavelength scanning multifunction reader (Tecan Infinite Pro, Switzerland) at excitation wavelength 748 nm and emission wavelength 750 nm, and the loading rate and entrapment efficiency of PLGA were calculated.

### SDS-PAGE Coomassie Brilliant Blue Analysis

To detect whether the MPD nanomaterial is coated with the cell membrane, the obtained MPD material was centrifuged, the underlying precipitated particles were taken, and the cell membrane was detected by the Coomassie Brilliant Blue method. According to the protein size, 10% separation glue and 5% concentrate glue were allocated. The processed samples were added to each sample well (the loading amount was 50 μg/well), and 10 μL protein marker was added to one of the wells. The concentration glue was electrophoresed at 80 V, and the separation glue was electrophoresed at 100 V. After electrophoresis, the gel was removed and the excess parts of the concentrated gel and separation gel were discarded. The gel was stained with Coomassie Brilliant Blue dye until the gel, the dye was fused (about 1 h) and decolorized with decolorizing solution for 24 h until the gel appeared as a clear blue protein band, and then the gel image was taken with Amersham Imager 600.

## *In Vitro* Experiment

### Cytotoxicity Study (Live/Dead Analysis)

LLC cells were cultured in confocal culture dishes with densities of 4 × 10^5^ cells/well and 2 × 10^5^ cells/well, respectively. The cells were cultured for 12 h and co-incubated with PBS, 1 mg/mL M-PLGA, and 1 μg/ml free DiR and nanocomposite materials containing the same DiR (PD and MPD) for 24 and 48 h, respectively. The calcein/PI cell activity and cytotoxicity detection kit was used to stain the living/dead cells according to the instructions, and the staining effect of the cells was detected by laser confocal microscopy. Note that the whole process should be operated without light.

### Capillary Cell Imaging Experiment

LLC cells were inoculated in cell culture dishes of 60 mm × 15 mm with a density of 5 × 10^5^ cells/dish for 24 h. After co-incubation for 1, 6, and 12 h with free DiR and nanocomposite materials (PD and MPD) containing the same amount of DiR, the cells were digested with trypsin and collected by centrifugation and then resuspended with 200 μL 2% paraformaldehyde. After the cell suspension was put into the imaging capillary tube and sealed at both ends, the cells were photographed by a LAZR-X multimode imaging system, and the whole process was done in the dark.

## *In Vivo* Experiments

### Establishment and Imaging of LLC Nude Mouse Model of Lung Cancer Tumor

The animal experiments were approved by the Institutional Animal Care and Use Committee of Guangzhou Medical University. About 4 × 10^6^ LLC cells were collected and inoculated subcutaneously into the right shoulder of BALB/c nude mice to establish a tumor-bearing mouse model. When the tumor volume reached 100 ± 10 mm^3^, the tumor-bearing mice were randomly divided into four groups (PBS, DiR, PD, and MPD) and were injected with the same amount of saline (200 μL), DiR (200 μL, 1 mg/ml), and PD and MPD containing the same amount of DiR through the tail vein. At 1, 4, 8, and 24 h after injection, mice were anesthetized with 2% isoflurane, and then the tumor fluorescence signal and PA imaging were monitored by the Vevo LAZR-X multimode imaging system.

After 24 h of imaging, the tumors and organs (the heart, liver, spleen, lung, and kidney) were dissected for fluorescence signal detection and PA imaging, respectively.

### Circulation Lifetime Evaluation

Sixteen BALB/c nude mice fasted overnight and were randomly divided into four groups (PBS, DiR, PD, and MPD). They were injected with the same amount of saline (200 μL), DiR (200 μL, 1 mg/ml), and PD and MPD containing the same amount of DiR through the tail vein. At 1, 2, 4, 6, 8, and 24 h after injection, 60 μL of blood was taken from the orbital venous plexus immediately, and 10 μL of anticoagulant was added and then mixed and stored at 4°C in the dark. The residual nanoparticles in the blood (μg) were calculated by the standard curve, and the curve of the percentage of the retention amount of nanoparticles in the blood to the initial injection amount, that is, the blood retention amount of nanoparticles (%), and the blood retention time was plotted.

## Data Availability

The original contributions presented in the study are included in the article/Supplementary Material, and further inquiries can be directed to the corresponding authors.

## References

[B1] Chang Z.-m. Zhang R. Yang C. Shao D. Tang Y. Dong W.-f. (2020). Cancer-leukocyte Hybrid Membrane-Cloaked Magnetic Beads for the Ultrasensitive Isolation, Purification, and Non-destructive Release of Circulating Tumor Cells. Nanoscale 12 (37), 19121–19128. 10.1039/d0nr04097e 32929419

[B2] Chen H.-Y. Deng J. Wang Y. Wu C.-Q. Li X. Dai H.-W. (2020). Hybrid Cell Membrane-Coated Nanoparticles: A Multifunctional Biomimetic Platform for Cancer Diagnosis and Therapy. Acta Biomater. 112, 1–13. 10.1016/j.actbio.2020.05.028 32470527

[B3] Chen Y. Zhang Y. Zhuang J. Lee J. H. Wang L. Fang R. H. (2019). Cell-membrane-cloaked Oil Nanosponges Enable Dual-Modal Detoxification. ACS Nano 13 (6), 7209–7215. 10.1021/acsnano.9b02773 31117372

[B4] Chong W. K. Papadopoulou V. Dayton P. A. (2018). Imaging with Ultrasound Contrast Agents: Current Status and Future. Abdom. Radiol. 43 (4), 762–772. 10.1007/s00261-018-1516-1 29508011

[B5] Dehaini D. Wei X. Fang R. H. Masson S. Angsantikul P. Luk B. T. (2017). Erythrocyte-platelet Hybrid Membrane Coating for Enhanced Nanoparticle Functionalization. Adv. Mater. 29 (16), 1606209. 10.1002/adma.201606209 PMC5469720 28199033

[B6] Franc B. L. Acton P. D. Mari C. Hasegawa B. H. (2008). Small-animal SPECT and SPECT/CT: Important Tools for Preclinical Investigation. J. Nucl. Med. 49 (10), 1651–1663. 10.2967/jnumed.108.055442 18794275

[B7] Gao L. Wang H. Nan L. Peng T. Sun L. Zhou J. (2017). Erythrocyte Membrane-Wrapped pH Sensitive Polymeric Nanoparticles for Non-small Cell Lung Cancer Therapy. Bioconjug. Chem. 28 (10), 2591–2598. 10.1021/acs.bioconjchem.7b00428 28872851

[B8] Hariri A. Zhao E. Jeevarathinam A. S. Lemaster J. Zhang J. Jokerst J. V. (2019). Molecular Imaging of Oxidative Stress Using an LED-Based Photoacoustic Imaging System. Sci. Rep. 9, 11378. 10.1038/s41598-019-47599-2 31388020 PMC6684596

[B9] Hu C.-M. J. Zhang L. Aryal S. Cheung C. Fang R. H. Zhang L. (2011). Erythrocyte Membrane-Camouflaged Polymeric Nanoparticles as a Biomimetic Delivery Platform. Proc. Natl. Acad. Sci. 108 (27), 10980–10985. 10.1073/pnas.1106634108 21690347 PMC3131364

[B10] Kroll A. V. Fang R. H. Zhang L. F. (2017). Biointerfacing and Applications of Cell Membrane-Coated Nanoparticles. Bioconjug. Chem. 28 (1), 23–32. 10.1021/acs.bioconjchem.6b00569 27798829 PMC5471317

[B11] Lee D.-E. Koo H. Sun I.-C. Ryu J. H. Kim K. Kwon I. C. (2012). Multifunctional Nanoparticles for Multimodal Imaging and Theragnosis. Chem. Soc. Rev. 41 (7), 2656–2672. 10.1039/c2cs15261d 22189429

[B12] Li H. Peng Q. Yang L. Lin Y. Chen S. Qin Y. (2020). High-performance Dual Combination Therapy for Cancer Treatment with Hybrid Membrane-Camouflaged Mesoporous Silica Gold Nanorods. ACS Appl. Mater. Inter. 12 (52), 57732–57745. 10.1021/acsami.0c18287 33326211

[B13] Li R. He Y. Zhang S. Qin J. Wang J. (2018). Cell Membrane-Based Nanoparticles: a New Biomimetic Platform for Tumor Diagnosis and Treatment. Acta Pharmaceutica Sinica B 8 (1), 14–22. 10.1016/j.apsb.2017.11.009 29872619 PMC5985624

[B14] Liu Y. Ai&Lu K. L. H. Lu L. (2012). Nanoparticulate X-ray Computed Tomography Contrast Agents: From Design Validation to *In Vivo* Applications. Acc. Chem. Res. 45 (10), 1817–1827. 10.1021/ar300150c 22950890

[B15] Peng Q. Li H. Deng Q. Liang L. Wang F. Lin Y. (2021). Hybrid Artificial Cell-Mediated Epigenetic Inhibition in Metastatic Lung Cancer. J. Colloid Interf. Sci. 603, 319–332. 10.1016/j.jcis.2021.06.066 34186407

[B16] Upputuri P. K. Pramanik M. (2020). Recent Advances in Photoacoustic Contrast Agents for *In Vivo* Imaging. WIREs Nanomed Nanobiotechnol 12 (4), e1618. 10.1002/wnan.1618 32027784

[B17] Wang J. Jeevarathinam A. S. Humphries K. Jhunjhunwala A. Chen F. Hariri A. (2018). A Mechanistic Investigation of Methylene Blue and Heparin Interactions and Their Photoacoustic Enhancement. Bioconjug. Chem. 29 (11), 3768–3775. 10.1021/acs.bioconjchem.8b00639 30281976 PMC8046596

[B18] Wang X. Xie X. Ku&Wang G. L. H. V. Wang L. V. Stoica G. (2006). Noninvasive Imaging of Hemoglobin Concentration and Oxygenation in the Rat Brain Using High-Resolution Photoacoustic Tomography. J. Biomed. Opt. 11 (2), 024015. 10.1117/1.2192804 16674205

[B19] Zhang L. Deng S. Zhang Y. Peng Q. Li H. Wang P. (2020b). Homotypic Targeting Delivery of siRNA with Artificial Cancer Cells. Adv. Healthc. Mater. 9 (9), 1900772. 10.1002/adhm.201900772 32181988

[B20] Zhang Y. Qin Y. Li H. Peng Q. Wang P. Yang L. (2020a). Artificial Platelets for Efficient siRNA Delivery to clear “Bad Cholesterol”. ACS Appl. Mater. Inter. 12 (25), 28034–28046. 10.1021/acsami.0c07559 32469502

