# Supplementary figures and images for: A Novel Biomimetic Nanoprobe as a Photoacoustic Contrast Agent
Source: Front Chem. 2021 Aug 3;9:721799. doi: 10.3389/fchem.2021.721799 (PMC8369425; doi:10.3389/fchem.2021.721799)

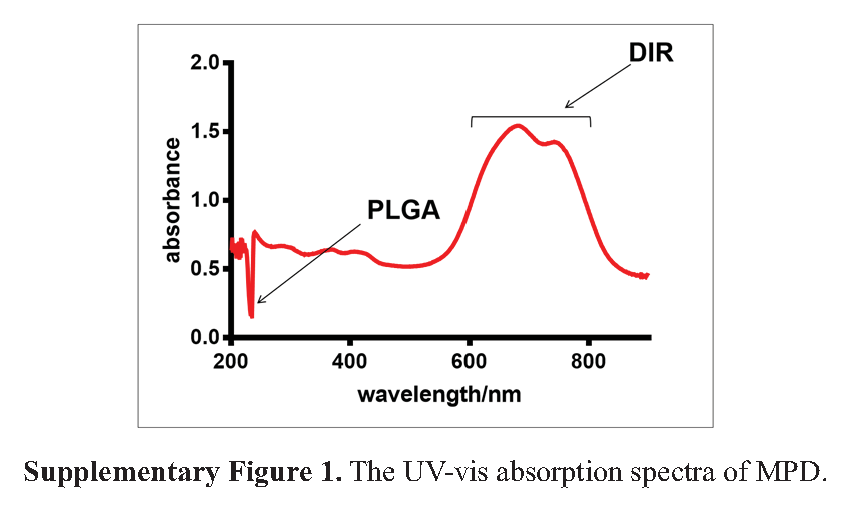

Supplement: Supplementary file 1 [file Image1.tiff]

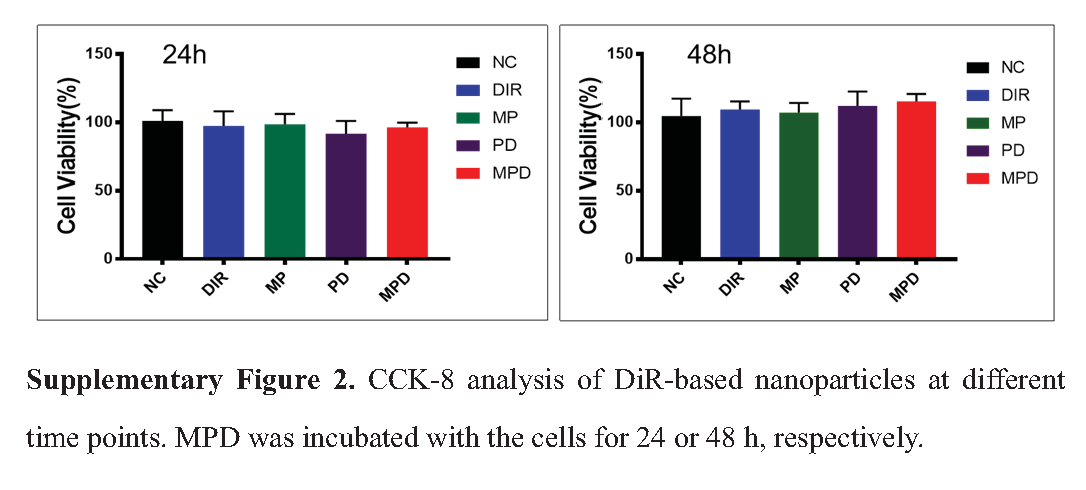

Supplement: Supplementary file 2 [file Image2.tif]
